# Heavy Metals Bioaccumulation and Health Risks with Associated Histopathological Changes in Clarias gariepinus from the Kado Fish Market, Abuja, Nigeria

**DOI:** 10.5696/2156-9614-10.26.200602

**Published:** 2020-05-04

**Authors:** Samson E. Abalaka, Simon I. Enem, Idoko S. Idoko, Nuhu A. Sani, Oremeyi Z. Tenuche, Sunday A. Ejeh, Wonder K. Sambo

**Affiliations:** 1 Department of Veterinary Pathology, University of Abuja, Abuja, Nigeria; 2 Department of Veterinary Public Health and Preventive Medicine, University of Abuja, Abuja, Nigeria; 3 Department of Veterinary Physiology and Biochemistry, University of Abuja, Abuja, Nigeria; 4 Veterinary Teaching Hospital, Ahmadu Bello University, Zaria, Nigeria

**Keywords:** Clarias gariepinus, aquatic pollution, heavy metals bioaccumulation, health risk assessment, histopathological changes

## Abstract

**Background.:**

Aquatic pollution by heavy metals has become a global problem and is of great concern due to the detrimental effects on aquatic ecosystems and possible human uptake.

**Objectives.:**

The present study aimed to evaluate tissue bioaccumulations of heavy metals and possible human health risks in addition to associated cellular damages in fish for sale in a fish market in Abuja, Nigeria.

**Methods.:**

Clarias gariepinus sold at the Kado Fish Market in Abuja, Nigeria, in March–April 2017 and March–April 2018 were randomly purchased and sampled. The heavy metal contents of the sampled fish were assayed and used to assess the extent of potential health risks to human consumers. The extent of histopathological changes associated with the bioaccumulations in the sampled fish were also evaluated according to standard procedures.

**Results.:**

Levels of most heavy metals were above permissible limits, except for lower levels of zinc (Zn) in liver and muscle and higher levels of lead (Pb) in muscle in the sampled fish. Similarly, significant (p<0.05) liver bioaccumulation occurred for Zinc (Zn), chromium (Cr), iron (Fe), and copper (Cu) compared to their levels in muscle. The total hazard index (non-cancer risk across all metals) was 0.0415, which is very low and acceptable. The cancer risks ranged between the acceptable values of 1.98 × 10^−5^ – 3.71 × 10^−6^. Associated histopathological changes, which occurred in the sampled fish, differed between the various assayed tissues.

**Conclusions.:**

Most of the heavy metals were significantly bioaccumulated (p<0.05) and were above the permissible levels, particularly in liver samples. The levels of muscle heavy metal bioaccumulation in the sampled fish posed no apparent cancer or non-cancer health risks to human consumers. However, further efforts are needed to minimize heavy metal aquatic environmental pollution to safeguard the health of aquatic flora and fauna and human consumers because of the tendency of these pollutants to persist and bioaccumulate to toxic levels over time.

**Competing Interests.:**

The authors declare no competing financial interests.

## Introduction

Aquatic environments are progressively exposed to heavy metals pollution due to increasing natural processes and anthropogenic activities. Although heavy metals may be essential or non-essential nutrients, they can be toxic at certain levels.^1^,^2^ Heavy metals are not biodegradable, and therefore may build-up, persist and move up aquatic food chains to threaten fish and pose health risks to human consumers. Fish can be used to determine aquatic ecosystem pollution due to their different sensitivities to water pollution, especially heavy metal pollution, as they are located at the top of the aquatic food chain.^3^,^4^ Heavy metal bioaccumulation in fish can give insights into the long-term status of aquatic environments.^5^

Levels of some heavy metals exceed international limits in water, fish, soils, edible vegetables, and food animals in many African countries.^6^ Although reports of heavy metals build-up in fish in Nigeria are well documented, most of this research has focused on the levels and sequence of bioaccumulation in fish in the wild.^7^–^9^ Few reports have addressed health risks to human consumers of these fish.^10^,^11^ Although Igwemmar *et al*., Abdulrahman *et al*. and Kelle *et al*. have documented the presence of heavy metals in fish sold in some Nigeria markets, information on the human health risks and associated histopathological changes in such fish is not readily available.^12^–^14^ This information is essential, as deaths have been recorded in fish with excessively bioaccumulated heavy metals above permissible levels, due to induced structural and functional changes.^1^,^15^–^17^ However, heavy metals toxicity varies significantly within and between fish species primarily due to structural disruptions arising from their ability to interfere with cellular enzyme-mediated processes.^18^ Therefore, determination of the levels of heavy metal bioaccumulation in fish is of particular interest globally not only because of the potential detrimental effects on fish, but also their eventual effects on consumers.^19^ Such investigations can be performed at either the source point (aquaculture) or at the end-user point (fish markets) to help safeguard the health status of both fish and consumers.

Although extensive research has been done on heavy metal bioaccumulation in fish, most of this research has been on fish muscle, and to a lesser extent on the gills, kidney, and liver, even though fish muscle is not always a good indicator of whole fish body contamination compared to the gills and liver.^20^–^24^ This may be due to anatomical locations and functional differences between these fish tissues, especially as fish muscle is the most commonly consumed part of the fish. Fish liver is the detox center as well as a reflector of heavy metal contamination and bioaccumulation in the entire fish body. Most of the freshwater fish offered and sold to the public in Nigerian fish markets are from commercial sources rather than from wild environments, and as such, they can be used to highlight levels of heavy metals pollution of their respective aquatic environments. However, heavy metal determination of their respective aquatic environments is practically impossible to accomplish, as the fish, supplied from different geographical areas and sources to these markets, are usually mixed and sorted into varying sizes before being offered for sale to the public. Most of the fish are sourced from concrete/tarpaulin or earthen ponds with appropriate environmental conditions and feeding regimens. Fish farmers harvest fish with the use of efficient pond water discharge or with nets, depending upon the size of the earthen pond. The fish are then gathered and sorted based on size in metal bathtubs.

The present work aimed to assess levels of heavy metal bioaccumulation and their associated histopathological changes in fish for sale in Nigerian fish markets (end-user point) using fish muscle bioaccumulations to determine health risks to consumers. The present study investigated levels of heavy metals build-up in the liver and muscles of Clarias gariepinus sold at the Kado Fish Market, Life Camp, Abuja, Nigeria, in relation to potential health risks to consumers, and associated histopathological effects in the sampled fish. C.gariepinus was chosen because it is one of the most commonly cultivated and consumed freshwater fish in Nigeria in general, and in Abuja in particular, due to its robust hardiness to harsh environmental conditions, choice taste and wide acceptance.

**Abbreviations**
*DTC*: Degree of tissue change
*THQ*: Target hazard quotient
*TR*: Target cancer risk

## Methods

In the present study, a total of 36 live adult Clarias gariepinus (962.90 ± 38.78 g mean weight and 50.46 ± 0.78 cm mean total length) were randomly purchased and sampled during two weeks in March–April of 2017 and March–April 2018 from the Kado Fish Market, Life Camp, Abuja, Nigeria. Fish were sampled without regard to age, sex or aquatic environmental sources, because the fish for sale in the market are usually supplied from different sources but are pulled together and sorted into various sizes, as different fish sizes command different prices in the market. In addition, fish are not tagged for ease of identification to trace them back to their respective aquatic sources for evaluation. The sampling periods coincided with the traditional Easter celebrations when fish supply and consumption is usually high in this part of the country. Gills, liver, kidney, and spleen tissues of the sampled fish were harvested for histopathological evaluation, while samples for heavy metals analysis were preserved on ice and transported to the laboratory for prompt analysis.^25^,^26^

### Heavy metals analysis

The present study assayed a total of six heavy metals comprised of four essential metals (zinc (Zn), iron (Fe), chromium (Cr), and copper (Cu)) and two non-essential metals (cadmium (Cd) and lead (Pb)) in the liver and muscles of sampled fish. An atomic absorption spectrophotometer (iCE 3000, Thermo Scientific, USA) was used for the assay based on Beer-Lambert's law, as described by Van Loon and Lichwa, following aqua regia digestion.^27^,^28^ Similarly, the accuracy and precision of the atomic absorption spectrophotometer was determined with a metal detection limit of 0.0001 using the spike recovery method in the absence of the use of standard materials as described by the International Union of Pure and Applied Chemistry.^29^ Briefly, metal recovery was performed by spiking the first portion of the homogenous solution of samples with 10 ppm of heavy metal analytes, while the second portion served as the un-spiked control. The amount of spiked metal analytes recovered after digestion of all samples was used to calculate the percentage recovery of each metal as described by Burns *et al*. using Equation 1.^30^


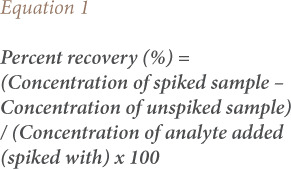


### Human health risk assessment

Target hazard quotient (THQ) and hazard index protocols were used to assess the non-carcinogenic risk posed by the consumption of these heavy metals in muscles of sampled fish according to the United States Environmental Protection Agency (USEPA), using Equations 2 and 3.^31^

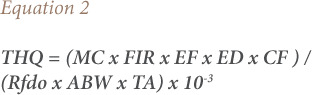
where, MC is the heavy metal concentration in the muscles of the fish sample (mg/kg); FIR is the fish ingestion rate, 20.8 g/day was used due to a 7.6 kg per capita fish consumption in Nigeria; EF is the exposure frequency (365 days/year); ED is the exposure period, which is equivalent to 53.1 years, the average life expectancy in Nigeria; CF is the conversion factor (0.208) used to convert fish fresh weight to fish dry weight considering a 79% moisture content of fish; Rfdo is the reference dose of individual heavy metal intake (mg/kg/day) as follows: Zn − 0.3, Cr − 0.003, Fe − 0.7, and Cu − 0.04, Cd − 0.001, and Pb − 0.004; ABW is the average body weight of 60 kg for an adult in Nigeria; and TA is the average exposure time for non-carcinogens (equivalent to EF × ED or 19381.5 according to Wang *et al.*). 32–36


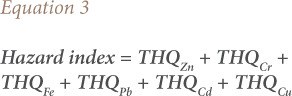


A THQ < 1 indicates an unlikely lifetime non-carcinogenic health risk to the human consumers of the exposed fish, while a THQ ≥ 1 indicates an increasing potential lifetime non-carcinogenic health risk to human consumers of the exposed fish.

Similarly, the target cancer risk (TR) or the potential risk of cancer development in humans over a lifetime of heavy metals exposure from exposed fish (muscles or flesh) as described by USEPA was determined using Equation 4.^31^

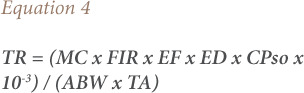
where, MC, FIR, EF, ED, ABW, and TA as described in Equation 2; and CPso is the carcinogenic potency slope of the metals: Pb − 0.0085, Cd − 0.38, and Cr − 0.5.^37^ An acceptable range is TR 10^−6^ – 10^−4^, TR ≤ 10^−6^ is considered inconsequential and TR ≥ 10^−3^ is considered to be of high potency, according to Wang *et al*.^36^

### Histopathological evaluation

We evaluated the gills, liver, spleen, and kidneys of the sampled fish for histopathological changes after harvest by fixing these organs in 10% neutral buffered formalin and promptly prepared and processed them according to standard procedures described by Bancroft and Cook.^38^ The extent of histopathological changes in these tissues was determined semi-quantitatively by adapting the degree of tissue change (DTC) method of Poleksic and Mitrovic-Tutundzic as modified by Camargo and Martinez using the formula: DTC = (1 × ***∑****I*) + (10 × ***∑****II*) + (100 × ***∑****III*), after establishing tissue lesions in stages classified as *I*, *II*, and *III* for each organ.^39^,^40^ Lesions that did not affect the normal functioning of the organ were considered as stage *I* alterations, stage *II* were those that impaired functioning and stage *III* lesions caused irreparable tissue damages. Likewise, the obtained values were interpreted as follows: normal functioning organ (0 – 10), slightly damaged organ (11 – 20), moderately damaged organ (21 – 50), severely damaged organ (51 – 100), and irreversibly damaged organ (> 100).

### Statistical analysis

The incidence of histopathological changes were expressed in simple percentages, including the level of heavy metals bioaccumulation as mean values (± standard error of the mean), which was further subjected to Mann-Whitney test for statistical significance (p<0.05) using the GraphPad software program (GraphPad Prism, version 6, USA).

## Results

**Figure 1:**
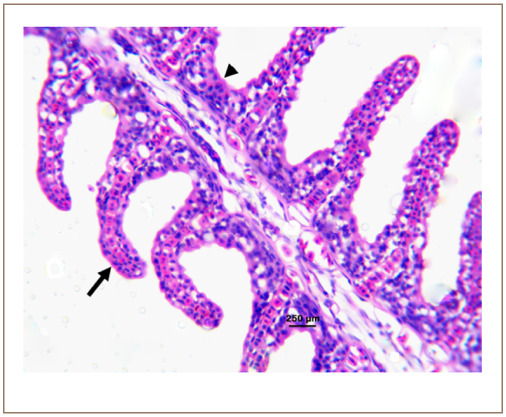
Photomicrograph of the gill of Clarias gariepinus sold at Kado Fish Market, showing the primary lamellar (arrowhead) and secondary lamellar (arrow). Hematoxylin and eosin, bar = 250 μm.

We obtained an 82.30 – 112.84% metal recovery range for all the assayed heavy metals based on the spike recovery protocol. Table 1 shows the liver and muscle levels of heavy metals in C. gariepinus sold at the Kado Fish Market, Life Camp, Abuja, Nigeria. The cancer and non-cancer risks to human consumers of sampled fish arising from bioaccumulation are shown in Table 2. Similarly, the incidence and DTC in the gills, liver, kidney, and spleen of the sampled fish are presented in Tables 3 and 4 based on the observed histopathological changes in affected tissues *(Figures 1 through 10).* Table 5 shows the effects of Pb and Cd bioaccumulation above and below the permissible level of DTC in Clarias gariepinus sold at the Kado Fish Market, Life Camp, Abuja, Nigeria. Similarly, Table 6 shows the effect of fish size on heavy metals bioaccumulation in the sampled fish.

**Figure 2:**
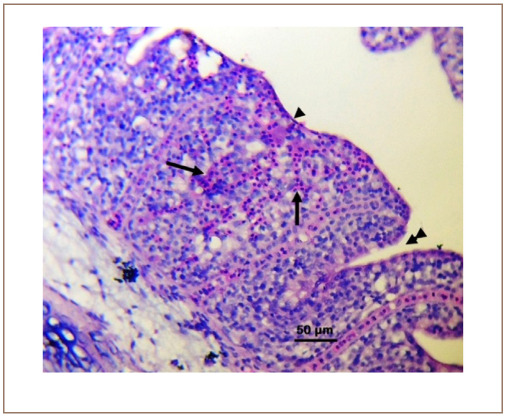
Photomicrograph of the gill of Clarias gariepinus sold at the Kado Fish Market, showing the complete lamellar fusion (arrowhead), partial lamellar fusion (double arrowheads) and lamellar hemorrhages (arrows). Hematoxylin and eosin, bar = 50 μm.

**Figure 3:**
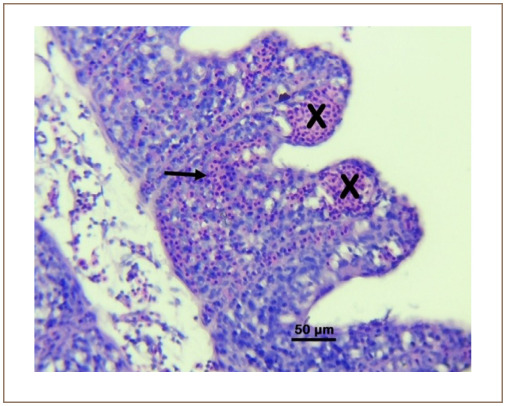
Photomicrograph of the gill of Clarias gariepinus sold at the Kado Fish Market, showing the lamellar aneurysms (X) and lamellar hemorrhage (arrow). Hematoxylin and eosin, bar = 50 μm.

**Figure 4:**
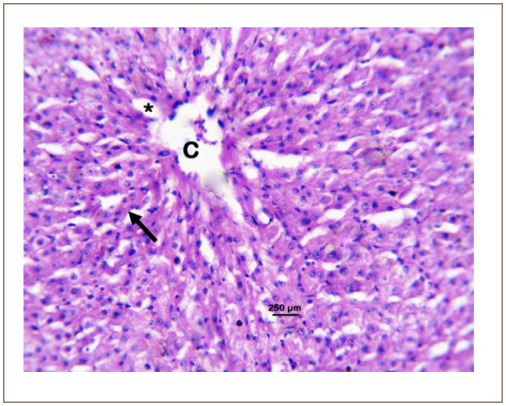
Photomicrograph of the liver of Clarias gariepinus sold at the Kado Fish Market, showing the central vein (C), hepatic cord (arrow) and sinusoid (*). Hematoxylin and eosin, bar = 250 μm.

**Figure 5:**
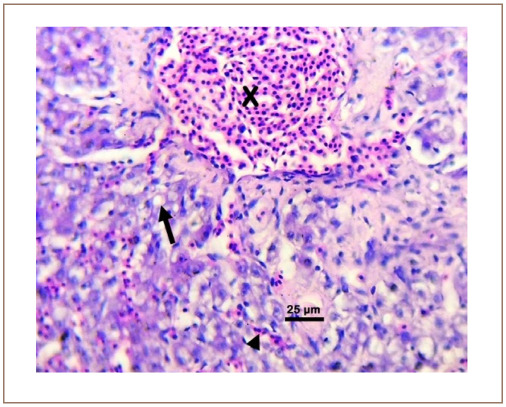
Photomicrograph of the liver of Clarias gariepinus sold at the Kado Fish Market, showing the congested central vein (X), congested sinusoid (arrowhead) and hepatic intracytoplasmic vacuolation (arrow). Hematoxylin and eosin, bar = 25 μm.

**Figure 6:**
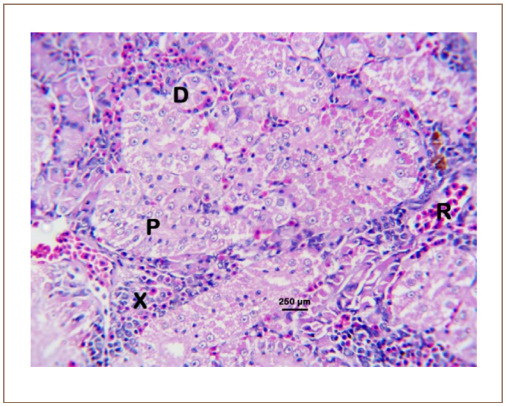
Photomicrograph of the kidney of Clarias gariepinus sold at the Kado Fish Market, showing the proximal convoluted tubule (P), distal convoluted tubule (D), hematopoietic tissue (X), and the renal vein (R). Hematoxylin and eosin, bar = 250 μm.

**Figure 7:**
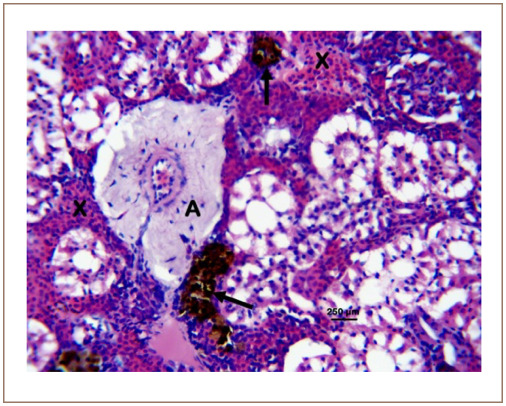
Photomicrograph of the kidney of Clarias gariepinuss sold at the Kado Fish Market, showing the thickened vascular wall (A), interstitial hemorrhages (X) and melano-macrophage centers (arrows). Hematoxylin and eosin, bar = 250 μm.

**Figure 8:**
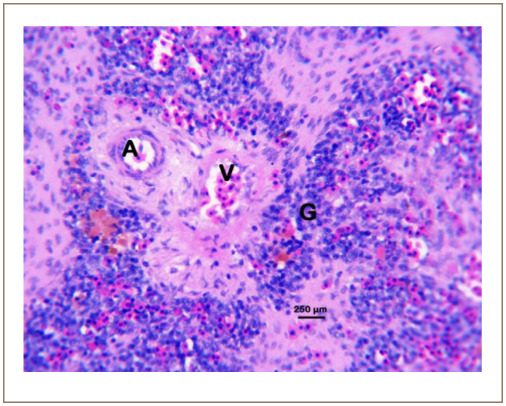
Photomicrograph of the spleen of Clarias gariepinus sold at the Kado Fish Market, showing the splenic artery (A), splenic vein (V) and the periarteriolar sheath (G). Hematoxylin and eosin, bar = 250 μm.

**Figure 9:**
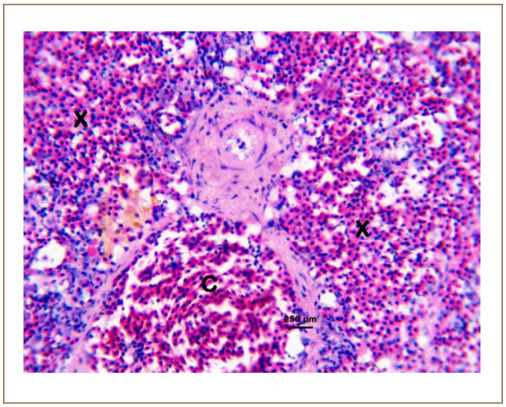
Photomicrograph of the spleen of Clarias gariepinus sold at the Kado Fish Market, showing the congested splenic vein (C) and congested parenchyma (X). Hematoxylin and eosin, bar = 250 μm.

**Figure 10:**
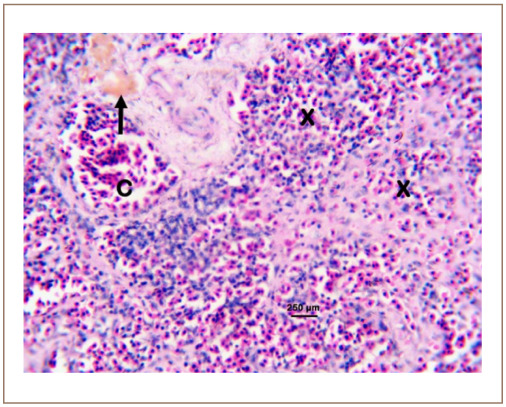
Photomicrograph of the spleen of Clarias gariepinus sold at the Kado Fish showing the congested splenic vein (C), depleted white blood cells pulp (X) and melano-macrophage center (arrow). Hematoxylin and eosin, bar = 250 μm.

**Table 1:** Heavy Metal Concentrations in Liver and Muscles of Clarias gariepinus Sold at Kado Fish Market

**Heavy metals**	**Permissible level (mg/kg)^41^**	**Heavy metal concentration (mg/kg)**		

		**Muscle**	**Liver**	**P-value**
Zinc	67.10	2.64 ± 0.05	22.46 ± 1.60	<0.0001*
Chromium	0.73	0.34 ± 0.01	4.63 ± 1.20	0.0139*
Iron	146.00	3.08 ± 0.07	221.90 ± 32.76	<0.0001*
Lead	0.12	1.26 ± 0.08	6.92 ± 2.21	0.7318
Cadmium	0.19	0.15 ± 0.05	0.23 ± 0.11	0.6771
Copper	3.28	0.23 ± 0.02	21.33 ± 2.49	<0.0001*

*Values with asterisks within rows shows liver levels differed significantly (p<0.05) from muscle levels.

**Table 2:** Human Risk Assessment of Consumption of Clarias gariepinus Sold at Kado Fish Market

**Heavy metals**	**Target hazard quotient**	**Target cancer risk**
Zinc	0.0007	-
Chromium	0.0082	5.89 × 10^−5^
Iron	0.0004	-
Lead	0.0228	3.71 × 10^−6^
Cadmium	0.0109	1.98 × 10^−5^
Copper	0.0005	-

Hazard index	0.0415	-

**Table 3:** Incidence and Stages of the Degree of Tissue Changes in Organs of Clarias gariepinus Sold at Kado Fish Market

**DTC stage**	**Degree of histopathological tissue changes**			

	**Gill alterations (%)**	**Liver alterations (%)**	**Kidney alterations (%)**	**Spleen alterations (%)**
*I*	EPD (19.44%) LED (25.00%) PLF (30.56%) MCI (2.78%)	VAC (19.44%) MCI (44.44%) TVW (27.78%) PBD (8.33%) FED (2.78%) MMC (2.78%)	VAC (2.78%) MCI (58.33%) TVW (30.56%) MMC (38.89%)	VAC (13.89%) MCI (2.78%)
*II*	CLF (50.00%) ANM (27.78%) HMR (16.67%)	CGT (77.78%) HMR (8.33%) BGM (2.78%)	CGT (50.00%) HMR (47.227%)	CGT (66.67%) MMC (66.67%)
*III*		HNE (5.6%)		LNE (69.44%)

Abbreviations: ANM, aneurysm; BGM, bile pigment; CGT, congestion; CLF, complete lamellar fusion; DTC, degree of tissue change; EPD, epithelial detachment; FED, fibrin exudation; HMR, hemorrhage; HNE, hepatic necrosis; LED, lamellar edema; LNE, lymphocytic necrosis; MCI, mononuclear cellular infiltration; MMC, melano-macrophage center; PBD, proliferated bile ducts; PLF, partial lamellar fusion; VAC, vacuolation; TVW, thickened vascular wall.

**Table 4:** Degree of Tissue Changes in the Gills, Liver, Kidney, and Spleen of Clarias gariepinus Sold at Kado Fish Market

**Tissues**	**Histopathological changes**	

	**Degree of tissue change**	**Interpretation**
Gills	8.94 ± 1.58	Normal function
Liver	10.22 ± 1.14	Slight damage
Kidneys	14.42 ± 1.56	Slight damage
Spleen	82.78 ± 8.75	Severe damage

**Table 5:** Effects of Lead and Cadmium Bioaccumulation Above and Below the Permissible Level on the Degree of Tissue Change in Clarias gariepinus Sold at Kado Fish Market

**Fish tissue**	**Degree of tissue change**					

	**Lead**			**Cadmium**					

	**Below permissible level (mg/kg)^41^**	**Above permissible level (mg/kg)^41^**	**P-value**	**Below permissible level (mg/kg)^41^**	**Above permissible level (mg/kg)^41^**	**P-value**
Gills	13.55 ± 2.89	8.12 ± 1.18	0.0693	8.10 ± 2.06	8.77 ± 1.24	0.5336
Liver	11.09 ± 2.16	10.07 ± 0.86	0.8200	10.30 ± 1.18	10.21 ± 0.91	0.5678
Kidney	8.46 ± 2.96	15.49 ± 1.13	0.0306*	14.80 ± 2.98	14.34 ± 1.19	0.3741
Spleen	54.55 ± 15.63	87.87 ± 6.53	0.0102*	94.00 ± 13.43	80.97 ± 13.43	0.7929

*Values with asterisks within rows shows above the permissible level differed significantly (p<0.05) from the below the permissible level.

**Table 6:** Effects of Fish Size on Heavy Metals Bioaccumulation in Clarias gariepinus Sold at Kado Fish Market

**Description**	**Permissible level (mg/kg)^41^**	**Tissue**	**Heavy metals (mg/kg) per fish body weight (g)**		

			**Large sized fish**	**Small sized fish**	**P-value**
Body weight (g)	-	-	1131.00 ± 40.09	794.60 ± 35.40	<0.000†
Zinc	67.10	Muscle	2.58 ± 0.06	2.70 ± 0.08	0.3055
		Liver	21.08 ± 2.36	24.02 ± 2.12	0.4076
Chromium	0.73	Muscle	0.34 ± 0.02	0.34 ± 0.02	0.9991
		Liver	2.02 ± 0.86	7.58 ± 2.16	0.0227*
Iron	146.00	Muscle	2.93 ± 0.10	3.23 ± 0.10	0.0194*
		Liver	160.40 ± 43.58	291.00 ± 44.66	0.0073*
Lead	0.12	Muscle	1.21 ± 0.12	1.31 ± 0.10	0.6881
		Liver	7.72 ± 3.48	6.02 ± 2.72	0.1993
Cadmium	0.19	Muscle	0.26 ± 0.10	0.04 ± 0.01	0.3038
		Liver	0.17 ± 0.10	0.30 ± 0.20	0.2359
Copper	3.28	Muscle	0.24 ± 0.02	0.21 ± 0.02	0.4056
		Liver	20.46 ± 2.85	22.31 ± 4.32	0.8713

*Values with asterisks within rows shows small-sized fish heavy metals bioaccumulation differed significance (p<0.05) from the large-sized fish heavy metals bioaccumulation.

## Discussion

The obtained values based on the spike recovery protocol were within the acceptable spike recovery range of 70.00 – 125.00% according to Machado and Griffith, which validated the digestion and atomic absorption spectrophotometer analysis methods used in the present study.^42^ Significant (p<0.05) liver bioaccumulation occurred in Zn, Cr, Fe, and Cu compared to their muscle levels, with insignificantly (p>0.05) higher Pb and Cd liver levels over muscle concentrations. The higher recorded liver bioaccumulations compared with muscles of the sampled fish agreed with earlier reports by Amundsen *et al*. and Rosli *et al*.^2^,^43^

The lower concentrations in muscle might be because fish muscles are not external boundary structures in direct contact with polluted waters. Similarly, unlike the liver where metals are readily mobilized and actively detoxified, fish muscles are not active detoxification centers. The obtained sequence of heavy metal build-up in the liver and muscles of the sampled fish differed from those recorded by Igwemmar *et al*.^12^ All liver heavy metals, excluding Zn, as well as muscle Pb, were bioaccumulated above the permissible levels in the sampled fish according to the International Atomic Energy Agency (IAEA), indicative of increasing aquatic environmental pollution.^41^ Although humans do not usually consume fish liver, the level of liver heavy metal bioaccumulation is considered a good biomonitor of aquatic pollution. This is because their levels of bioaccumulation in fish organs are often proportional to levels present in the environment.^16^ Although the present study did not assay for mercury (Hg) levels in the sampled fish, Kelle *et al.* reported 0.109 – 1.233 ppm of Hg in the tissues of C. gariepinus sold in Asaba major markets in Delta State, Nigeria.^14^ They reported some tissue bioaccumulation above the 0.5 ppm permissible level, according to the Food and Agriculture Organization of the United Nations (FAO).^44^ Nevertheless, most bioaccumulation studies in Nigerian fish samples have not involved Hg assay, highlighting the need for future investigations.^14^

Most of the heavy metals bioaccumulated more in smaller-sized fish compared to larger-sized fish, and significant (p<0.05) bioaccumulation occurred only in fish muscle (Fe) and fish liver (Cr and Fe) of the small-sized fish compared to those of the large-sized fish. This finding suggests that fish size might influence heavy metal bioaccumulation, especially for smaller-sized fish. Nevertheless, there are currently no definite or established relationships between fish size and heavy metal bioaccumulation, again highlighting the need for further investigations.^45^ Differences in the evaluated cancer and non-cancer risks to consumers of the sampled fish are attributable to differences in levels of individual and cumulative bioaccumulation of these heavy metals in the muscles of the sampled fish. Although Pb and Cd accounted for 54.94% and 26.26% of the hazard index in the sampled fish, the bioaccumulated heavy metals did not pose a lifetime non-carcinogenic risk to consumers of the sampled fish. Similarly, individual heavy metal TRs were all within the acceptable range, according to Wang *et al*.^36^ These findings also meant that there were no immediate cancer risks over a lifetime consumption of these sampled fish. Differences in bioaccumulation levels of these heavy metals could account for the varying levels of the observed cellular damages in the sampled fish because levels of bioaccumulations determine the extent of toxicity.

Observed gill lesions did not alter their functionality based on the DTC interpretation because the observed changes were mostly epithelial alterations rather than vascular changes, including cellular death, from which recovery is usually easier and faster.^39^ Abalaka reported similar gill alterations associated with heavy metal contamination of an aquatic environment.^17^ The adaptive nature of the observed alterations was responsible for the recorded low DTC in affected gills. Histopathological changes in the liver resulted in slight liver damage in the sampled fish. These histopathological changes, which were not metal-specific, are generally associated with hepatic responses to toxicant exposures.^46^ Such toxicants-induced cellular changes are often seen in the liver as consequences of their regular metabolic, detox and biotransformational activities.^47^ Similarly, histopathological changes in the affected kidneys also resulted in slight damage. These changes might not be unconnected with the kidney's excretory role that predisposes it to toxicant injuries. The present study recorded severe damage in the spleen of the sampled fish mostly in the form of lymphocytic depletion, which was probably due to lymphocytic necrosis that might result in immune-suppression.

The same normal, slight, and severe tissue damages in the gills, liver, kidney and spleen were recorded in fish with Pb and Cd bioaccumulation above and below their permissible levels. However, only slight gill damage and normal functioning kidney was recorded in fish with bioaccumulated Pb levels below the permissible level based on the DTC protocol. These findings showed that cellular damage occurred in fish with Pb and Cd bioaccumulation irrespective of their levels of bioaccumulation. This might be attributable to the toxicity of non-essential heavy metals like Pb and Cd when present even in trace amounts as well as the toxic effects of essential heavy metals like Zn, Fe, Cr, and Cu when present at high concentrations.^48^ The above notwithstanding, the possible cellular damaging effects of fish handling, transportation, and withholding could have also induced these lesions, according to Harper and Wolfe.^49^

No significant (p>0.05) variations existed in the DTC of fish with Pb and Cd levels above and below permissible limits, except in Pb-bioaccumulated fish where more significant (p<0.05) kidney and spleen tissue damage occurred compared to the damage in fish below the permissible Pb level. Overall, the greatest cellular damage occurred in the spleen. Recorded variations in the extent of cellular damage among the tissues of the sampled fish might be due to the propensity of heavy metals to induce multiple organ damage resulting from their individual or cumulative synergistic, additive or antagonistic effects.^50^,^51^

## Conclusions

Although some heavy metals bioaccumulated far above the permissible levels in the sampled fish, their effects posed a greater threat to fish health than immediate cancer and non-cancer health risks to their human consumers. However, care must be taken to reduce the levels of heavy metal bioaccumulation in fish sold at the Kado Fish Market because of heavy metals' ability to build up and bio-magnify over time. Effective legislation and proper implementation of appropriate industrial waste disposal measures can help to reduce heavy metal aquatic environmental pollution.
